# Gene profiling in white blood cells predicts infliximab responsiveness in rheumatoid arthritis

**DOI:** 10.1186/ar1990

**Published:** 2006-07-03

**Authors:** Thierry Lequerré, Anne-Christine Gauthier-Jauneau, Carine Bansard, Céline Derambure, Martine Hiron, Olivier Vittecoq, Maryvonne Daveau, Othmane Mejjad, Alain Daragon, François Tron, Xavier Le Loët, Jean-Philippe Salier

**Affiliations:** 1 CHU de Rouen, Hôpitaux de Rouen, Service de Rhumatologie, Rouen, F-76000, France; 2 Inserm, U519, Rouen, F-76000, France; 3 Université Rouen, Faculté de Médecine-Pharmacie, Institut Fédératif de Recherche Multidisciplinaire sur les Peptides, Rouen, F-76000, France; 4 Consortium EGERIE, Rouen, Paris, France

## Abstract

As indicators of responsiveness to a tumour necrosis factor (TNF)α blocking agent (infliximab) are lacking in rheumatoid arthritis, we have used gene profiling in peripheral blood mononuclear cells to predict a good versus poor response to infliximab. Thirty three patients with very active disease (Disease Activity Score 28 >5.1) that resisted weekly methotrexate therapy were given infliximab at baseline, weeks 2 and 6, and every 8th week thereafter. The patients were categorized as responders if a change of Disease Activity Score 28 = 1.2 was obtained at 3 months. Mononuclear cell RNAs were collected at baseline and at three months from responders and non-responders. The baseline RNAs were hybridised to a microarray of 10,000 non-redundant human cDNAs. In 6 responders and 7 non-responders, 41 mRNAs identified by microarray analysis were expressed as a function of the response to treatment and an unsupervised hierarchical clustering perfectly separated these responders from non-responders. The informativeness of 20 of these 41 transcripts, as measured by qRT-PCR, was re-assessed in 20 other patients. The combined levels of these 20 transcripts properly classified 16 out of 20 patients in a leave-one-out procedure, with a sensitivity of 90% and a specificity of 70%, whereas a set of only 8 transcripts properly classified 18/20 patients. Trends for changes in various transcript levels at three months tightly correlated with treatment responsiveness and a down-regulation of specific transcript levels was observed in non-responders only. Our gene profiling obtained by a non-invasive procedure should now be used to predict the likely responders to an infliximab/methotrexate combination.

## Introduction

Rheumatoid arthritis (RA) is a chronic, auto-immune and inflammatory polyarthritis that induces joint damage and disability. Tumour necrosis factor (TNF)α plays a key role in the associated pathological events and has been identified as a therapeutic target. In fact, TNFα blocking agents (TBAs), such as infliximab, etanercept, and adalimumab, have revolutionized the therapeutic care of methotrexate-resistent patients. Various clinical trials with a TBA/methotrexate combination have shown efficacy in 60% to 80% of such patients [1-3].

TBAs reduce joint inflammation, slow down joint damage and improve physical function [4,5]. Still, 20% to 40% of the RA patients given a TBA/methotrexate combination do not respond to this treatment [1-3]. Moreover, TBAs may have side effects and are costly [6] and the efficacy of any given TBA in a given patient is unpredictable [7,8]. For these reasons, predicting responsiveness to a given TBA or other emerging biotherapies (such as inhibitors of the interleukin-1 or interleukin-6 pathways) would be most useful. Markers that have proven informative for RA diagnosis or prognosis, such as C-reactive protein (CRP), erythrocyte sedimentation rate, autoantibodies (for example, rheumatoid factors and anti-cyclic citrullinated peptide antibodies), metalloproteinases and bone proteins cannot predict the responsiveness to TBAs [9].

Because genetic polymorphisms such as HLA-DR haplotypes have been associated with a variable natural course of RA and a heterogeneous response to conventional disease-modifying anti-rheumatic drugs (DMARDs), a few studies have attempted to identify genetic markers for TBA efficacy and they have focused on the promoters of several cytokine genes [10-12]. For example, sequence variation in the *TNFα *gene promoter has been associated with a variable response to infliximab [11]. However, similar conclusions hold true for etanercept as well [13] and, therefore, such genotypings are useless for selecting the TBA with greatest benefits [14].

Because response to treatment likely depends on polymorphisms at multiple loci [15], genome-wide analysis of gene expression with cDNA arrays has been recently used to identify markers of responsiveness in the peripheral blood mononuclear cells (PBMCs). However, the number of such studies is still very limited [16,17] and very few informative genes have been identified [16]. Moreover, in all instances, too few patients per study precluded statistically valid conclusions [17] or a confirmatory analysis in another, independent set of patients [16].

Owing to transcriptome analysis in PBMCs from RA patients, we have now identified a small subset of transcripts whose combined levels allow one to reliably predict the response to a infliximab/methotrexate combination in methotrexate-resistant patients with very active disease.

## Materials and methods

### Patients

A total of 33 patients, fulfilling the American College of Rheumatology (ACR) criteria for RA [18] and followed in Rouen University Hospital were included in this study. The criteria for patient eligibility were: methotrexate treatment; disease activity score 28 (DAS28) = 5.1 [19]; and resistance to at least one DMARD (methotrexate included). Exclusion criteria were: evolving infectious disease; age <18 years; no contraception; pregnancy; cancer less than 5 years old; cardiac failure (stage III-IV of the New York Heart Association); and infliximab allergy. This protocol (numbered 2003/007) was approved by the ethics committee of Haute-Normandie (France) and all participants signed an informed consent at the time of enrolment. For one month or more before the start of this study every patient was given fixed amounts of a DMARD and nonsteroidal anti-inflammatory drug (NSAID) and did not receive any intra-articular steroid injections. During this study, every patient was given the same doses of methotrexate and prednisone as used before, and was treated with infliximab (Remicade^®^, Schering-Plough, Levallois Perret, France) as recommended by the manufacturer and the French Drug Agency AFSSAPS (intravenous 3 mg/kg infliximab at weeks 0, 2, 6, and every 8th week thereafter). Before each infiximab infusion, DAS28, plasma CRP level, patient's assessment of pain (0 to 100 mm visual analogue scale), duration of morning stiffness, and physical function scored with the French version of the Health Assessment Questionnaire for RA [20] were recorded. Just before the 4th infusion (that is, at 3 months), the patients were categorized as responders whenever a change of DAS28 = 1.2 was obtained. All others were categorized as non-responders.

### PBMC isolation and mRNA extraction and labelling

The PBMCs were isolated from venous blood by Ficoll-Hypaque centrifugation and total RNAs were extracted by a standard phenol/chloroform procedure, quality controlled on an Agilent 2100 Bioanalyzer (Agilent Technologies, Palo Alto, USA) and frozen at -80°C until further use. An internal, arbitrary standard was made of a mixture of total RNAs from PBMCs taken from three healthy donors. The oligodT-primed poly(A) mRNAs were labelled with [α^33^P]dCTP as previously described [21], and the resulting, labelled cDNAs were immediately used for hybridisation.

### Transcriptome analysis and qRT-PCR

Our array covering 12,000 cDNA probes for 10,000 non-redundant genes and various negative controls as well as nylon arraying of PCR-amplified probes and hybridisation of [α^33^P]dCTP-labelled mRNAs have all been extensively described and validated in a previous report [21]. Briefly, cDNA probes selected on the basis of a tissue-preferred expression in liver corresponded to genes with a liver-restricted expression (10% of the probes) as well as genes with an hepatic expression along with a broad expression in some (50%) or many non-hepatic tissues (40%) [21]. All arrays were made from a single batch of cDNA probes. Every RNA sample was hybridised at least twice on separate arrays. Whenever necessary, the sequence of cDNA probes was controlled with an ABI3100 capillary sequencer (Applied Biosystems APPLERA-France, Courtaboeuf, France). Real-time, quantitative reverse transcription PCR (qRT-PCR) of mRNAs and normalization with the 18S RNA amount were done in duplicate as described [21]. The primers designed with the Primer3 software [22] are listed in Table 1.

### Image analysis and data mining

Image analysis with the XDotsReader software, version 1.8 (COSE, Le Bourget, France), subtractions of noise and spot background, and image normalization with the median value of all signals per image were done exactly as previously detailed [21]. A transcript was considered to be expressed if at least two hybridisations provided a positive signal. The resulting, normalized values were used for a selection of significantly regulated mRNAs, that is, those with an abundance that differed in two or more comparisons between two samples, using a funnel-shaped confidence interval (*p *< 0.05) calculated from every mRNA detected per hybridisation [21]. This results in a false discovery rate that is below 10% of the total number of regulated mRNAs. Statistical analyses were done with the R software [23]. The TIGR Multiexperiment viewer (Tmev version 2.2) [24] was used for unsupervised hierarchical clustering (HC) using the average dot product and complete linkage options, the leave-one-out cross-validation, and the supervised statistical tool Significance Analysis of Microarrays (SAM) for identification of discriminant transcripts [25] with a false discovery rate set at <1%. Information about our clinical and experimental data complies with the recommendations for the minimum information about microarray experiments (MIAME) and the raw data have been deposited (accession number GSE3592) in the GEO repository [26].

## Results

### RA patients and response to treatment

We categorized patients into two groups, responders (R1 to R16) and non-responders (NR1 to NR17) to an infliximab/methotrexate combination, at three months according to the EULAR criteria, as recommended [18]. Tables 2 and 3 provide demographic and clinical information for these 33 patients, at entry and at 3 months. The average disease duration was 11 to 12 years and the DAS28 score indicated that all these patients had a high level of RA activity, which fits with their resistance to one or more DMARDs. Before treatment, three variables (morning stiffness, DAS28, CRP level) were slightly different in responders compared to non-responders. Following treatment, the DAS28 score significantly improved at 3 months in responders (average decrease 2.3) whereas it remained high in non-responders (average decrease 0.4). Patients in both groups were randomly separated into either a training subset (subset 1) for transcriptome analysis or a validation subset (subset 2) for qRT-PCR. At this stage, we paid attention to retaining a relatively large number of patients in subset 2 of both groups. As noted in Tables 2 and 3, most features did not significantly differ between the paired subsets 1 and 2.

### Gene profiling in pre-treatment PBMCs correlates with treatment responsiveness

**Figure 1:**
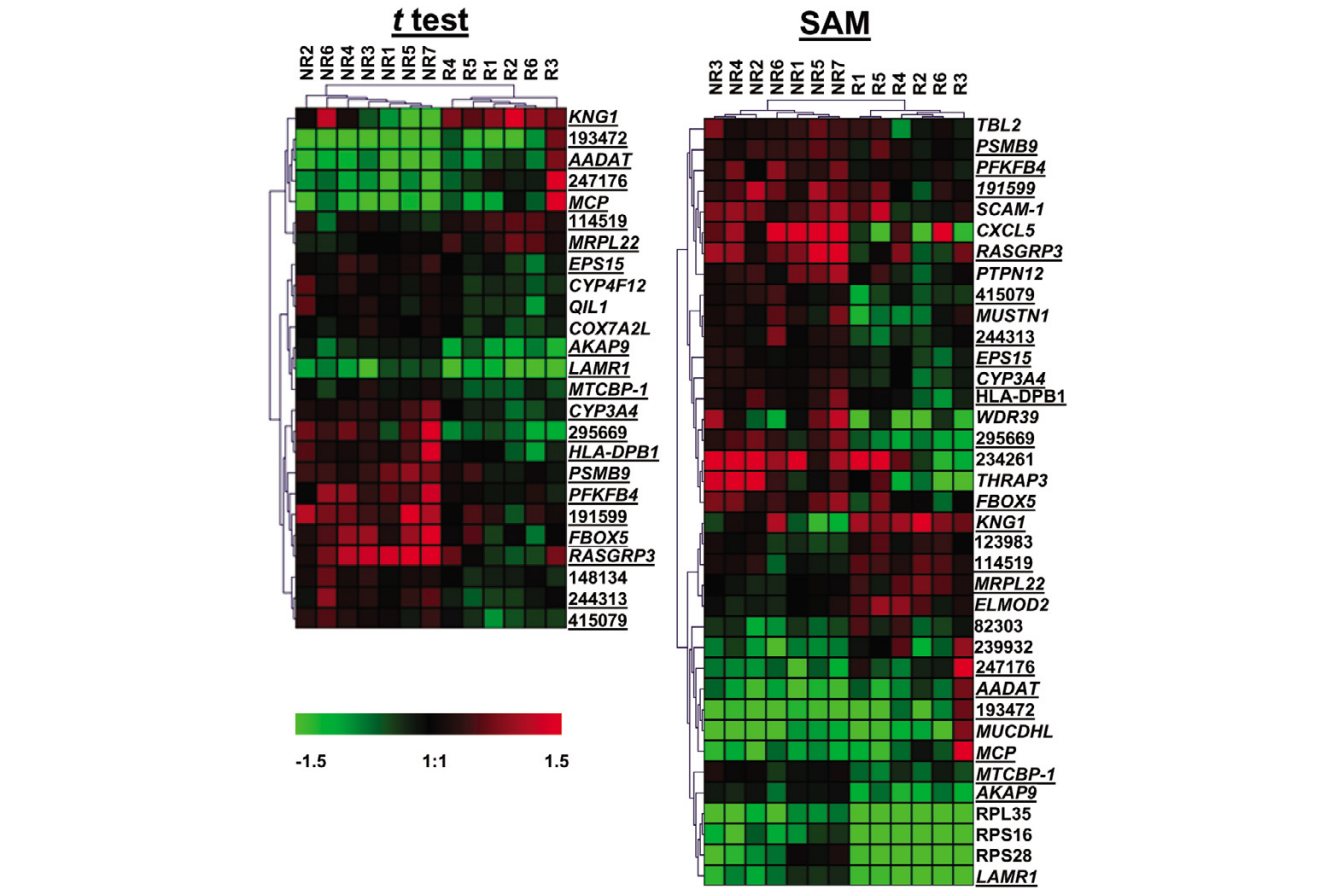
Clustering of rheumatoid arthritis patients as responders versus non-responders. Transcripts in peripheral blood mononuclear cells from six responders (R) or seven non-responders (NR) who were included in two training subsets (subset 1 in text and Tables 2 and 3) were studied by microarray analysis. Informative transcripts as selected by a statistical analysis (*t *test, 25 transcripts; significance analysis of microarrays (SAM), 37 transcripts) were next used for an unsupervised hierarchical clustering of the same 13 patients, listed as columns. The gene names are listed as rows (expressed sequence tags are noted with a plain, five to six digit IMAGE clone number). The genes are underlined whenever they were selected by both SAM and *t *test. Transcript levels are expressed as ratios (level in sample/level in internal, arbitrary standard). Scale bar (log2 ratio): decreased (green), increased (red) or identical (black) ratio in sample versus standard (grey squares are missing values).

Gene profiling in PBMCs was studied in the two training subsets (subset 1) of the responders and non-responders groups (a total of 13 patients). On average, 5,282 ± 1,253 transcripts were detected in PBMCs, with 86% overlap in transcript identities between responders and non-responders (data not shown). To precisely identify the transcripts that were differentially regulated in responders compared to non-responders, we first selected every transcript whose level in at least one responder was significantly different from the median value in non-responders or vice versa. This was assessed with a funnel-shaped confidence interval (see Materials and methods; *p *< 0.05) and resulted in 2,239 transcripts with an abnormal level in at least 1 out of these 13 patients. From these 2,239 transcripts, we next selected every transcript whose variation between responders and non-responders was statistically significant according to a *t *test (25 transcripts) and/or SAM (37 transcripts); these transcripts are listed in Figure 1 (total, 41 transcripts; overlap between *t *test and SAM selections, 21 transcripts) and detailed in Table 4. The identity of the corresponding microarray cDNA probes was verified by sequencing. Finally, we performed an unsupervised hierarchical clustering of the 13 patients above (subset 1). This was based on the levels of the 25 or 37 transcripts indicated above that, in both instances, resulted in a perfect separation of the responders and non-responders into two major clusters (Figure 1).

**Figure 2:**
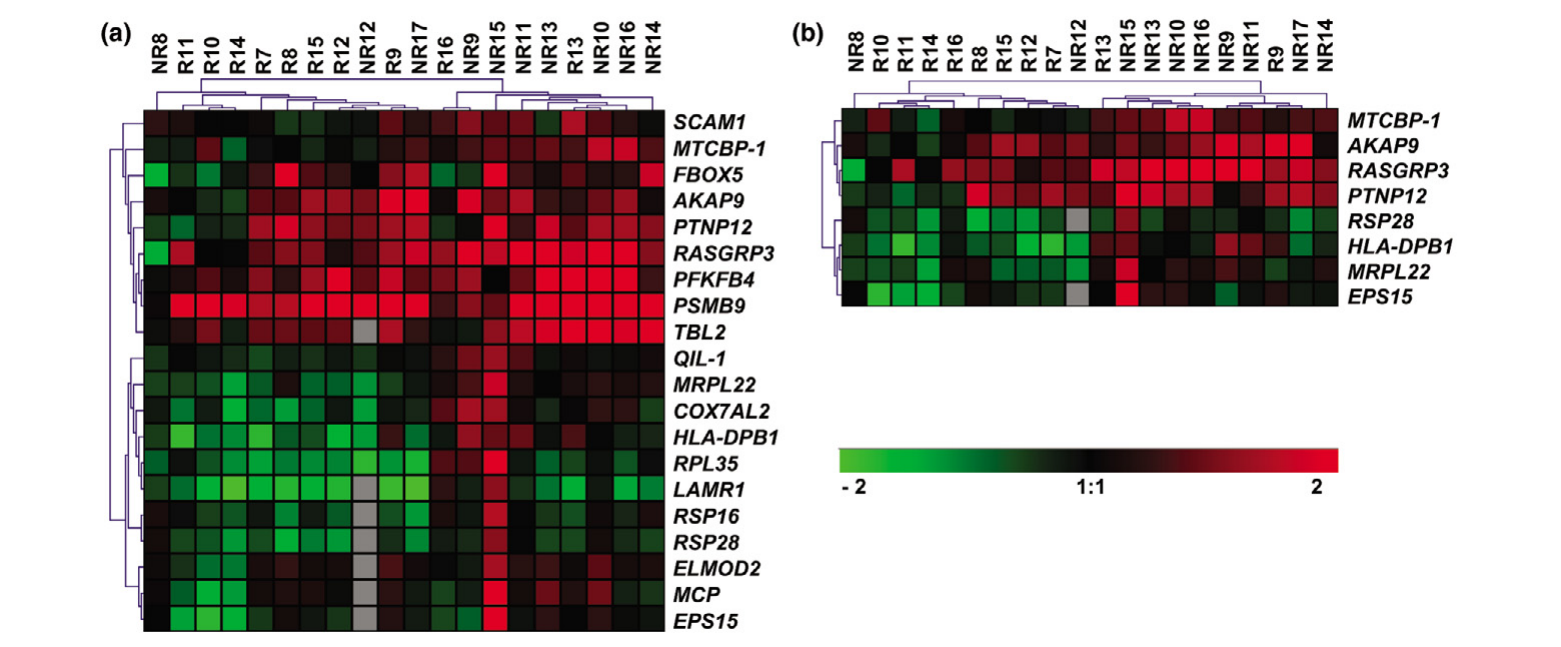
Validation of a narrow selection of transcripts as a tool for clustering responders versus non-responders. Ten responders (R) and ten non-responders (NR) were included in two validation subsets (subset 2 in text and Tables 2 and 3). In any given sample of peripheral blood mononuclear cells, the abundances of informative transcripts were determined by qRT-PCR and normalized with the corresponding 18S RNA level. Unsupervised hierarchical clusterings obtained with **(a) **20 or **(b) **8 selected transcripts are shown. Expression of transcript levels and scale bar are as in Figure 1.

We wished to confirm that a combination of the above transcript levels could be used as a predictor of responsiveness. For this purpose, we aimed to measure the levels of the above 41 transcripts by qRT-PCR and compare them between our two validation subsets (subset 2) from the responder and non-responder groups (a total of 20 patients). However, among these 41 transcripts, 12 putative transcripts were identified by only one IMAGE clone without knowledge of the intron/exon structure and, therefore, they were not retained. Moreover, among the 29 remaining transcripts, 9 of them failed to provide reliable data by qRT-PCR, despite repeated attempts with various primers. Eventually, 20 out of our 41 transcripts could be reliably quantified by qRT-PCR. As shown in Figure 2a, an unsupervised hierarchical clustering of the 20 patients in subset 2 from the two groups, as based upon these 20 transcript levels, resulted in two major clusters of responders versus non-responders, with 5 misclassified patients (NR8, NR12, NR17, R13, R16). Despite being informative, such a hierarchical clustering lacks statistical power, and the efficiency of the above set of 20 transcripts for patient classification was thus further evaluated by leave-one-out cross-validation [24]. This procedure identified 4 misclassified patients and indicated that this set of transcripts provides 90% sensitivity and 70% specificity for identification of responders and non-responders (Table 5).

To determine the minimal number of transcripts that should be measured for an acceptable prediction of responsiveness, we tested a series of combinations of transcripts in the 20 patients from each subset 2, and we varied the number and identity of the transcripts actually used (data not shown). With a given set of only 8 transcripts, 16 out of 20 patients could be correctly classified as responders or non-responders by hierarchical clustering (Figure 2b). Finally, leave-one-out cross-validation (Table 5) identified only two misclassified patients and indicated that a given set of 8 transcripts as a predictor of responsiveness was at least as accurate as the set of 20 transcripts above.

### Post-treatment transcript levels correlate with treatment responsiveness

**Figure 3:**
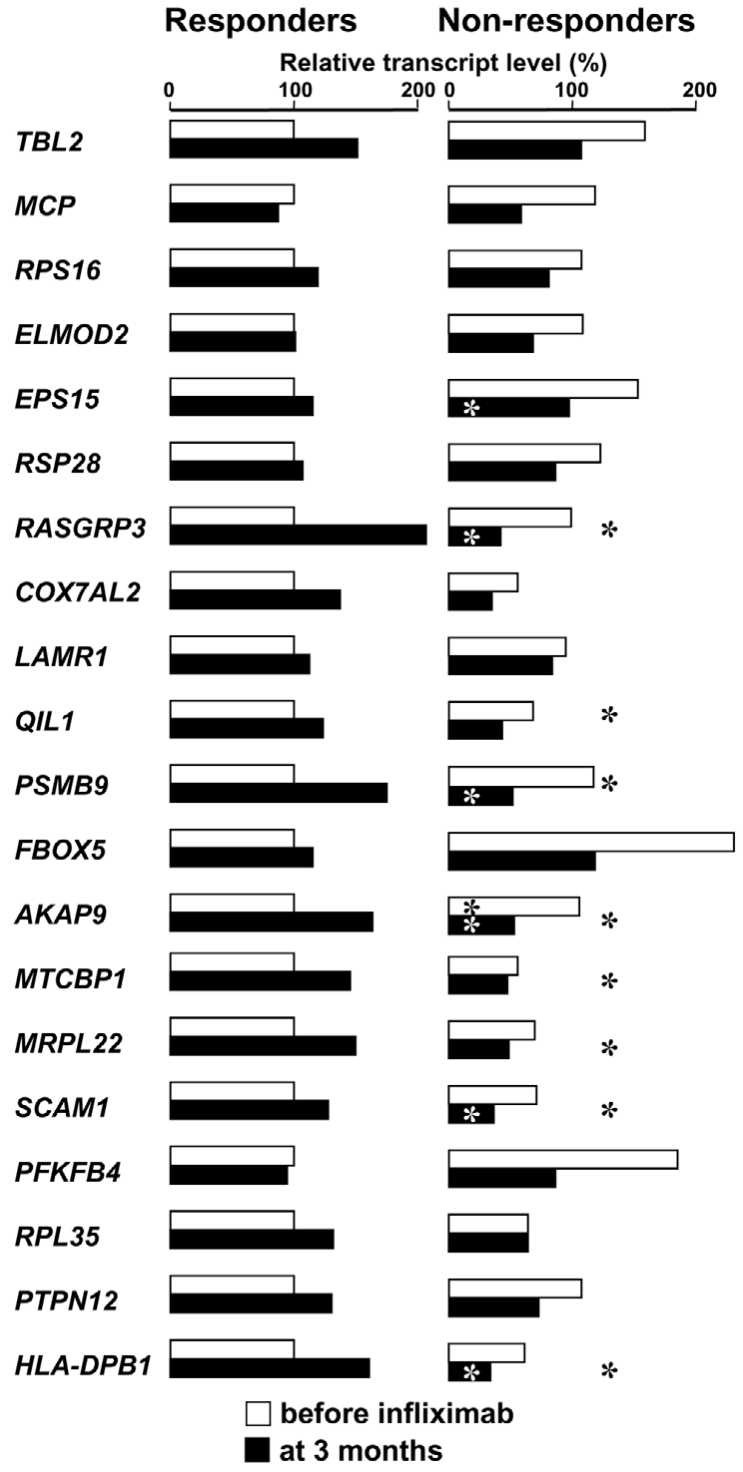
Relative transcript levels at baseline versus three months in responders or non-responders. The patients and transcripts are as in Figure 2a. For every transcript, the 4 levels (median value) shown at baseline and after 3 months in responders and non-responders are expressed as a percentage of the median level at baseline in responders (100%). Significant differences are all noted in the non-responder panel: asterisk outside closed bar, difference in non-responders at baseline versus three months (*p *< 0.05, paired Wilcoxon's test); asterisk within open bar, difference at baseline in responders versus non-responders (*p *< 0.05, Mann and Whitney's test); asterisk within closed bar, difference at 3 months in responders versus non-responders (*p *< 0.05, Mann and Whitney's test). In any patient group, a trend towards an increased or decreased level was considered whenever the value at 3 months was, respectively, above or below the value at baseline, whatever the difference of these values. Note that standard deviations are not shown because they are useless for non-parametric statistical tests.

We investigated whether the differences in transcript levels seen in responders compared to non-responders at baseline were also retained at three months. The data obtained by qRT-PCR with PBMCs are presented in Figure 3. In responders, 18 out of 20 transcripts (90%) exhibited a trend towards an increased level at 3 months, although the differences with respect to the levels at baseline were not significant. Strikingly, in non-responders, 19 out of 20 transcripts (95%) exhibited an opposite trend, that is, a decreased level at 3 months, and this difference was statistically significant for each of 8 transcripts (Figure 3). Overall, the differences in numbers of up- versus down-regulated transcripts in responders versus non-responders were highly significant, whether considering only the number of transcripts with a significant difference at baseline versus 3 months (*n *= 8, *p *= 3.10^-3^, Fisher's exact test) or considering the complete set of transcripts and associated trends (*n *= 20, *p *< 10^-4 ^by Fisher's exact test, or *p *= 0.007 by analysis of variance). This argues for a regulation of the corresponding genes by one (or more) TNFα-dependent pathway(s).

## Discussion

The small set of biological markers usually used for RA diagnosis or prognosis is unable to predict individual responsiveness to TBA [14]. Therefore, to enable such a prediction, global approaches based on proteomics or transcriptomics have been recently considered [27,28]. However, in the context of RA, proteomic analysis is still under development [27]. Moreover, very few informative transcripts have been identified by gene profiling [16] and the few studies that used this approach have relied on the differences in transcript levels measured at baseline versus two to three days after treatment onset [17]. This required exposure of every patient to treatment. Furthermore, the narrow time frame of this procedure may blur some significant but late variations with respect to baseline, which eventually limits transcript informativeness. In contrast, we have now measured transcript levels at baseline as the single predictor of responsiveness. In clinical practice, prediction can then be done without any exposure to treatment, which enables it to be restricted to responders.

Three months of treatment was chosen as the endpoint of our study, as recently recommended by international experts [29], because the objective of an efficient RA treatment is a rapid response. Should this early evaluation at three months disclose a moderate or absent response, this procedure allows another treatment to be used as early as possible. Also, using the DAS28 evolution at three months for classifying our 33 patients as responders or non-responders turned out to be quite reliable in the long run. Indeed, 22 out of 33 patients could be followed for three more years and their infliximab responsiveness, or lack thereof, did not vary over this period, even when increasing infliximab amount and frequency in non-responders (data not shown).

We aimed to identify a list of transcripts whose combined levels could be related to infliximab/methotrexate responsiveness. In fact, infliximab used alone is known to be efficient only for a short durationbecause the rapid production of anti-infliximab antibodies counteracts the drug's effect, whereas methotrexate advantageously limits this occurrence. The mixture of a cytokine inhibitor (infliximab) and an inhibitor of cell proliferation (methotrexate) is likely to regulate or even co-regulate a complex set of genes; this is a limitation if an understanding of some underlying events in RA is desired.

Gene expression was measured in PBMCs because this is an acknowledged, non-invasive procedure for diagnosis or prognosis of autoimmune diseases [30]. Specifically, in the context of RA, PBMCs as a surrogate tissue are advantageous as they allow for screening in any subject, whereas synovium is amenable to analysis in only a few patients. However, a drawback of such PBMC analysis is the lack of a clear-cut relationship between PBMCs and the affected synovium, which prevents the resulting data from providing an understanding of the RA-associated events in joints. Also, we analysed the PBMC transcriptome with an arbitrary collection of approximately 10,000 cDNA probes [21]. Since this restrictive procedure cannot measure every transcript expressed in the PBMCs, it does not intend to provide a genome-wide view of the RA-associated gene dysregulations in this tissue. Yet, this approach is quite acceptable when inferring prognosis from gene profiling is the major task.

Overall, the present study was not designed primarily to increase our understanding of RA physiopathology but is mostly suited to the predictive use of some combined transcript levels. Our data illustrate that a non-invasive transcriptome analysis done in PBMCs with an array of probes devoid of a specific selection towards the disease under study enables the efficient prediction of treatment responsiveness. Whether these conclusions are solid whatever the microarray/qRT-PCR platforms used, depend on a restricted PBMC subpopulation, or, above all, are useful in the context of an actual therapeutic decision, remains to be tested.

By *t *test and/or SAM, we identified a short list of 25 to 37 transcripts whose combined expression levels in PBMCs are an efficient discriminator of responders versus non-responders to infliximab/methotrexate. Many of the 25 transcripts identified by *t *test were no longer significant when using Bonferroni's correction to adjust statistics for the multiple transcripts analysed, but Bonferroni's correction has been recognized as a drastic one when used in this context, which contrasts with the SAM-associated false discovery rate [31]. Moreover, the *t *test and SAM cross-validated each other for most of the 20 transcripts eventually selected for qRT-PCR as 13 out of 20 (65%) such transcripts were significant with both tests (Table 4). Measuring these 20 transcript levels by qRT-PCR indicated that their performance as a predictor of responsiveness was equal to that obtained with 37 transcripts. Ultimately, a given combination of 8 selected transcripts (75% of them being significant by *t *test and SAM) as a predictor of responsiveness was as powerful as any higher number of transcripts. This observation that a given combination of very few transcripts can equal or even outperform the predictive strength of a higher number of transcripts has also been reported in another context, namely the response to hepatitis C treatment [32]. This small size for an informative gene set is most encouraging when the need comes for the development of a reliable, fast and cheap assay for measuring informative transcript levels in a clinical setting.

Consistent with the limitations noted above, our list of 29 transcripts did not disclose any significant series of transcripts whose altered levels could point to the physiopathological importance of a predominating function or pathway. Indeed, these transcripts covered such diverse proteins and functions as: ribosomal components (LAMR1, MRPL22, RPL35, RPS16, RPS28), which may suggest the existence of a TNFα-dependent pathway in the control of translation; cell adhesion and inhibition of cell migration/invasion (LAMR1, MUCDHL, MTCPB1); cytochromes (CYP3A4, CYP4F12) and cytochrome oxidase (COX7A2L); proteasome-mediated proteolysis (FBXO5, PSMB9); various enzymes (AADAT, PFKFB4); intra- or extracellular signalling (AKAP9, CXCL5, PTPN12, RASGRP3, TBL2, THRAP3), including regulators of the ERK pathway (EPS15, SCAM-1); and innate or adaptive immunity (KNG1, MCP, PSMB9, HLA-DPB1). Two transcripts, namely MUSTN1 and HLA-DPB1, are noteworthy; the MUSTN1 transcript codes for a protein involved in bone development and regeneration [33] and some alleles of the *HLA-DPB1 *gene have been associated with a relatively high risk of RA occurrence [34].

The opposite variations in transcript levels seen in responders compared to non-responders at three months strongly suggest that the informative transcripts retained in our study originated from TNFα-regulated genes. In fact, TNFα-dependent expression of the *CXCL5*, *CYP3A4*, *LAMR1*, *MCP*, and *PSMB9 *genes, as noted here, has been previously described [35-40]. However, only two of our transcripts, namely MCP and PTPN12, are found among lists of genes that are directly regulated by the TNFα/NFκB pathway, whether in RA [41] or in another context [42,43]. Therefore, it is likely that most of our transcripts are indirect TNFα targets. This view fits with the fact that the opposite variations in responders versus non-responders were observed weeks after the start of TBA. The reason why the transcript levels exhibited a limited trend to up-regulation at three months in responders along with a predominating repression in non-responders (Figure 3) also fits with indirect TNFα target genes, whose regulation would depend on one or more TNFα-dependent transcriptional repressor(s). The difference in responders versus non-responders could then result from genetic polymorphisms in binding sites for such repressors.

This situation of variations in binding of transcription factors has been previously described in RA [11,44]. Notably, the -308G/G genotype of the *TNFα *gene promoter is known to be associated with a better response to infliximab compared to the -308A/G or A/A genotype [11]. Other binding sites for repressor(s) could be located in any gene that belongs to the pathway from TNFα signalling to its indirect target genes whose transcripts were found here. If so, identifying such binding site polymorphisms that could predict the extent of responsiveness to TBA deserves further studies. Beyond this, it might well be worth combining the *HLA-DRB1 *genotype, itself a predictor of responsiveness to methotrexate/sulphasalazine/hydroxychloroquine in RA [45], with our measure of informative transcript levels, as this might enhance the predictive power of such indicators.

## Conclusion

The combined levels of a small set of discriminative transcripts have provided for the first time a tool for the prediction of infliximab/methotrexate efficacy in patients with long standing (11 to 12 years) and very active RA. It remains to be seen whether our predictive approach can prove useful in patients with recent and/or moderate RA activity or in non-responders given a higher dose of infliximab (>3 mg/kg). Other future studies should identify further gene profiles whose changes correlate with a responsiveness to other TBAs or treatments, such as interleukin-1 receptor antagonists [46]. Ultimately, we anticipate that a small series of parallel tests for such drug-specific combinations of transcripts, as quantified on a specifically designed DNA chip, should allow one to select the most appropriate treatment for every RA patient, with the resulting and beneficial eradication of the non-responder or moderate responder phenotypes.

## Abbreviations

CRP = C-reactive protein; DAS = disease activity score; DMARD = disease-modifying anti-rheumatic drug; PBMC = peripheral blood mononuclear cell; qRT-PCR = real-time, quantitative reverse transcription PCR; RA = rheumatoid arthritis; SAM = significance analysis of microarrays; TBA = TNFα blocking agent; TNF, tumour necrosis factor.

## Competing interests

A patent application EP 06290789.4 for the set of 20 or 8 transcripts with predictive power (Figure 2) has been deposited by Inserm. The authors declare that they have no competing interests.

## Authors' contributions

TL, XLL and JPS were responsible for designing the study and writing the manuscript. OV, OM, AD, and XLL were responsible for clinical coordination, access to samples, RA evaluation and manuscript improvements. ACGJ, CB, CD, and MH were responsible for microarrays, qRT-PCRs and data analysis. MD and FT provided numerous manuscript improvements.

## Figures and Tables

**Table 1:** Primers used for qualitative RT-PCR

Transcript	Forward	Reverse
AKAP9	5'-TGTTACTGGGTGGGTTCCAG-3'	5'-CAGAACCTGTGACTCGATGC-3'
COX7AL2	5'-TGATTTCCCTGGAGGTTCTG-3'	5'-CCCCGAGGTGACTAACTCAA-3'
ELMOD2	5'-AGCTCCTGCTCCCCCTAGTT-3'	5'-TCGCTGCAATTCACACTTCC-3'
EPS15	5'- GTCTTCCTTCCCCTCCCTTG-3'	5'-GCAGCATCAGAAGCCAACAC-3'
FBOX5	5'-CGCTGTAATTCACCTGCAAA -3'	5'-GTACCAGGCAGGGGACCTAT-3'
HLA-DPB1	5'-GACCTTCCAGATCCTGGTGA-3'	5'-CTTTCTTGCTCCTCCTGTGC-3'
LAMR1	5'- GCAGCAGGAACCCACTTAGG-3'	5'-AATGGCAACAATTGCACGAG-3'
MCP	5'-AGCAATTTGGAGCGGTAAGC-3'	5'-GTCCAGGTGCAGGATCACAA-3'
MRLP22	5'-CTCCACAACTGCCTGGAGAA-3'	5'-AACTGAGCCAAAGCCTGGTC-3'
MTCBP1	5'-GGAGAAGGGAGACATGGTGA-3'	5'-ACGAGGCACGTGTTAGTTCC-3'
PFKFB4	5'-TGGATCCCAAGTCCTTTGTG-3'	5'-CGCCTTGGACATCTCTTAGC-3'
PSMB9	5'-GGTTCTTGATTCCCGAGTGTC-3'	5'-CAGCCAAAACAAGTGGAGGT-3'
PTPN12	5'-TCCAGCGGGAGGTATTCACT-3'	5'-TGGTCCTTTGGGTTTTCCAC-3'
QIL1	5'-CCTCATCAAGGGAAGTGTGG-3'	5'-GGAGTCACGGATGGGAAAGT-3'
RASGRP3	5'-CAGCAAAGGGCAGAAGTCAT-3'	5'-TAATTGCCGTTGGAGGAGAC-3'
RPL35	5'-ACCTGAAGGTGGAGCTGTCC-3'	5'-AGAACACGGGCAATGGATTT-3'
RPS16	5'-AGTTCTGCTTCTCGGCAGG-3'	5'-TCTTGGAAGCCTCATCCACA-3'
RPS28	5'-GACCGGTTCTCAGGGACAGT-3'	5'-TGACTCCAAAAGGGTGAGCA-3'
SCAM1	5'-TGTGGCCCAGTACACCTTCA-3'	5'-CACGTAGCTGGCAGGGAATA-3'
TBL2	5'-GATGGGGGCTACACCTTCAC-3'	5'-TGACCCTTCAGGCTCCAGAT-3'
18S	5'-GTGGAGCGATTTGTCTGGTT-3'	5'-CGCTGAGCCAGTCAGTGTAG-3'

**Table 2:** Demographic and clinical data of rheumatoid arthritis patients at entry of study

Parameter	Responders^a^		Non-responders	
	Subset 1^b ^(*n *= 6)	Subset 2 (*n *= 10)	Subset 1 (*n *= 7)	Subset 2 (*n *= 10)

Age (years)	54.1 ± 13.8^c^	55.2 ± 9.2	56.1 ± 11.7	58.9 ± 11.6
Sex (men/women)	1/5	2/8	1/6	4/6
RA duration (years)	11.7 ± 8.0	11.1 ± 7.3	12 ± 10.2	10.5 ± 5.3
Methotrexate (mg/week)^d^	12.5 ± 5.5	13 ± 2.8	15.4 ± 2.7^e^	11.5 ± 3.2
Prednisone (mg/day)	12.1 ± 5.6	8.5 ± 4.2	10.3 ± 8.7	8.2 ± 5.4
Patients with NSAIDs	3	6	5	4
Patients with rheumatoid factor	4	8	5	6
Patients with anti-CCP abs^g^	3	8	5	8

^a^Categorised as indicated in Materials and methods. ^b^Transcript levels were measured by microarray analysis for subset 1 or qRT-PCR for subset 2. ^c^Mean ± standard deviation. ^d^Maximally tolerated dose in a given patient. ^e^Significant difference between subsets 1 and 2 within non-responders (*p* < 0.05, Mann and Whitney's non-parametric test). In this table, all other comparisons were non-significant. Anti-CCP abs, anti-cyclic citrullinated peptide antibodies; NSAID, non-steroidal anti-inflammatory drugs; RA, rheumatoid arthritis.

**Table 3:** Clinical data at baseline and at 3 months

	Responders				Non-responders			
	Subset 1		Subset 2		Subset 1		Subset 2	

	Baseline	3 months^a^	Baseline	3 months^a^	Baseline	3 months^a^	Baseline	3 months^a^

Morning stiffness (minutes)^b^	245 ± 126^c^	35 ± 24.5^c^	210 ± 81	58 ± 70.2^c^	179 ± 159	66.4 ± 86^c^	133 ± 84	62 ± 67.6^c^
DAS28 ^b^	6.4 ± 1.0	4.2 ± 0.9^c^	6.2 ± 0.7	3.8 ± 0.6^c^	5.7 ± 0.8	5.3 ± 1.0	5.5 ± 1.0	4.9 ± 1.0^c^
Pain (0–100 mm VAS)	59.3 ± 20.3	29.3 ± 9.3^c^	62.5 ± 15.5	31.3 ± 14.5^c^	69.3 ± 13.1	54.1 ± 22.1	60.9 ± 11.4	40.6 ± 18.4^c^
ESR (mm/hour)	44 ± 26.2	27 ± 20.3^c^	27.2 ± 15.7	11.3 ± 5.2^c^	35.7 ± 25.7	28.3 ± 15.3	24.1 ± 11.5	27.8 ± 19.2
CRP (mg/l)^b^	42 ± 29.8	20 ± 15.7^c^	28.6 ± 19.7	6.2 ± 6.1^c^	18.5 ± 12.7	13 ± 8.2	15.8 ± 15.6	11 ± 7.3
HAQ score (0–3 scale)	1.6 ± 0.4	0.9 ± 0.5^c^	1.8 ± 0.7	1.2 ± 0.7^c^	1.6 ± 0.4	1.2 ± 0.3	1.5 ± 0.4	1.5 ± 0.4

Values are mean ± standard deviation. ^a^Response assessed just before the fourth infliximab/methotrexate infusion. Significant differences between groups are as follows: ^b^difference between all responders versus non-responders at baseline (0.03 <*p *< 0.05, Mann and Whitney's test); other comparisons were non-significant (*p *= 0.58); ^c^Difference at baseline versus 3 months in this subset (*p *< 0.05, paired Wilcoxon's test). CRP, C-reactive protein; DAS, disease activity score; ESR, erythrocyte sedimentation rate; HAQ, health assessment questionnaire; VAS, visual analogue scale (patient's assessment of pain).

**Table 4:** Transcripts as predictors of infliximab responsiveness

IMAGE clone^a^	Encoded protein	Symbol^b^	Gene localisation	SAM^c^	*t *test^d^
295669	Clone 10PTELO13	-	-	-3.77	0.001
77684	CytP450, family 3, subfamily A, polypeptide 4	CYP3A4	7q21.1	-2.90	<10^-4^
417137	A kinase (PRKA) anchor protein 9	**AKAP9**	7q21-q22	-2.83	0.002
415079	Hypothetical protein DKFZp566M1046	-	-	-2.78	0.001
1848509	RP1 containing part of thyroid hormone receptor-associated protein 3	THRAP3	1p34.3	-2.56	NS
234261	RP11-750K11	-	-	-2.53	NS
198699	C-X-C chemokine ligand 5 (ENA78)	CXCL5	4q12-q13	-2.50	NS
730048	Ribosomal protein SA (37LRP)	**LAMR1**	3p21.3	-2.43	0.007
56923	F-box protein 5	**FBXO5**	6q25-q26	-2.42	0.006
1524020	RAS guanyl releasing protein 3 (calcium and DAG-regulated)	**RASGRP3**	2p25.1-p24.1	-2.41	0.004
756784	WD repeat domain 39	WDR39	2q11.2	-2.40	NS
244313	Bac clone RP11-576F1	-	-	-2.39	0.002
124452	6-Phosphofructo-2-kinase/fructose-2,6-biphosphatase 4	**PFKFB4**	3p22-p21	-2.33	0.003
724887	Major HLA, class II, DP beta 1	**HLA-DPB1**	6p21.3	-2.32	<10^-4^
416493	Ribosomal protein L35	**RPL35**	9q34.1	-2.25	NS
191599	Hypothetical protein FLJ13614	-	-	-2.23	0.006
726045	Ribosomal protein S16	**RPS16**	19q13.1	-2.24	NS
772993	Similar to 40S ribosomal protein S28	**RPS28**	19p13.2	-2.23	NS
110169	Proteasome subunit β type 9 (LMP2)	**PSMB9**	6p21.3	-2.17	0.006
346678	Musculoskeletal, embryonic nucleic protein 1	MUSTN1	3p21.1	-2.16	NS
741027	Vinexin β (SH3-containing adaptor molecule-1)	**SCAM-1**	8p21.3	-2.15	NS
428222	EGF receptor pathway substrate 15	**EPS15**	1p32	-2.12	0.003
740374	Transducin (beta)-like 2	**TBL2**	7q11.23	-2.12	Ns
774502	Protein tyrosine phosphatase, non-receptor type 12	**PTPN12**	7q11.23	-2.09	NS
320298	Membrane-type 1 matrix metalloprotein cytoplasmic tail binding protein 1	**MTCBP-1**	2p25.2	-2.04	0.005
148134	RP1-29K1 containing *KiAA0426*	-	-	NS	0.002
127203	CytP450, family 4, subfamily F, polypeptide 12	CYP4F12	19p13.1	NS	0.005
428560	QIL1 protein	**QIL1**	19p13.3	NS	0.009
810626	Cytochrome c oxidase subunit VIIa polypeptide 2 like	**COX7A2L**	2p21	NS	0.007
123983	Clone PR13	-	-	+1.80	NS
486624	ELMO domain containing 2	**ELMOD2**	4q31.21	+1.85	NS
114519	FLJ 14775	-	-	+1.90	0.007
357960	Mitochondrial ribosomal protein L22	**MRPL22**	5q33.1-q33.3	+1.99	0.009
82303	Hypothetical protein BC009264	-	-	+2.12	NS
247517	Mucin and cadherin-like	MUCDHL	11p15.5	+2.40	NS
194455	Membrane cofactor protein (CD46)	**MCP**	1q32	+2.30	0.005
247176	RP116103J18	-	-	+2.41	<10^-4^
195723	Kininogen 1	KNG1	-3q27	+2.46	0.009
239932	ELAC homolog 2	-	-	+2.46	NS
244896	Aminoadipate aminotransferase	AADAT	4q33	+2.52	0.002
193472	RP11-722P15	-	-	+2.68	0.002

^a^IMAGE clone number as a unique identifier. ^b^Bold indicates a transcript that was further tested by qRT-PCR. ^c^Significance analysis of microarrays (SAM) value as an indicator of significant transcript variation in responders versus non-responders. A positive or negative value indicates an over- or underexpression at baseline in responders versus non-responders, respectively. ^d^*P *value of a *t *test as an indicator of significant transcript variation in responders versus non-responders. NS, non- significant.

**Table 5:** Performance of the number of transcripts for prediction of responsiveness

	Number of selected transcripts^a^	
	20	8
Number of NR patients classified as NR^b^	7	10
Number of NR patients classified as R^b^	3	0
Number of R patients classified as R^b^	9	8
Number of R patients classified as NR^b^	1	2
Fisher's exact test^c^	*p *< 0.02	*p *< 0.0007
Sensitivity	90%	80%
Specificity	70%	100%
Positive predictive value	75%	100%
Negative predictive value	87.5%	83%

^a^As listed in Figure 2a, b. ^b^By leave-one-out cross-validation with 20 patients, including 10 non-responders (NR) and 10 responders (R) (referred to as validation subset 2 in the text). ^c^*P *< 0.05 indicates a significant link between transcript-based classification (R versus NR) and actual responsiveness.
